# Experimental reduction of host *Plasmodium* infection load affects mosquito survival

**DOI:** 10.1038/s41598-019-45143-w

**Published:** 2019-06-19

**Authors:** Rafael Gutiérrez-López, Josué Martínez-de la Puente, Laura Gangoso, Jiayue Yan, Ramón Soriguer, Jordi Figuerola

**Affiliations:** 1 Department of Wetland Ecology, Estación Biológica de Doñana (EBD-CSIC), Calle Américo Vespucio 26, E-41092 Seville, Spain; 2 Theoretical and Computational Ecology, Institute for Biodiversity and Ecosystem Dynamics, University of Amsterdam, Science Park, 904 1098 XH Amsterdam, The Netherlands; 3 Department of Ethology & Biodiversity Conservation, Estación Biológica de Doñana (EBD-CSIC), Calle Américo Vespucio 26, E-41092 Sevilla, Spain; 4 CIBER Epidemiología y Salud Pública (CIBERESP), Seville, Spain; 5 Present Address: Illinois Natural History Survey, University of Illinois, 1816 S Oak St., Champaign 61820 Illinois, USA

**Keywords:** Ecological epidemiology, Parasitology

## Abstract

*Plasmodium* transmission success depends upon the trade-off between the use of host resources to favour parasite reproduction and the negative effects on host health, which can be mediated by infection intensity. Despite its potential influence on parasite dynamics, the effects of infection intensity on both, birds and vectors, and on *Plasmodium* transmission success are still poorly understood. Here, we experimentally reduced the *Plasmodium* load in naturally infected wild house sparrows with the antimalarial primaquine to assess the effects of intensity of infection in the vertebrate hosts on *Plasmodium* transmission to and by mosquitoes. We monitored the survival of *Culex pipiens* mosquitoes throughout the development of the parasite and the infection status of the mosquitoes by analysing the head-thorax and saliva at 13 days post-exposure to birds. The proportion of mosquitoes infected by *Plasmodium* and the presence of *Plasmodium* in saliva were not associated with the medication treatment of birds. However, the experimental treatment affected vector survival with mosquitoes fed on medicated birds showing a higher survival rate than those fed on control individuals. These results provide strong experimental evidence of the impact of parasite load of vertebrate hosts on the survival probability of malaria vectors.

## Introduction

Parasites depend on their hosts to survive and to maximise their fitness^1^. Avian *Plasmodium* are vector-borne parasites that reproduce asexually in birds but requires mosquitoes to complete their sexual reproduction and development of sporozoites before being successfully transmitted. In mosquitoes, during sporogony, numerous sporozoites, i.e., parasite forms with elongated bodies, are formed in the oocyst. After maturation of the oocysts, the sporozoites move into the haemocoele and then penetrate the salivary glands of the vector. Transmission occurs when sporozoites, the infective forms of the parasites, are injected during a vector blood meal into the vertebrate hosts^2^. Thus, parasite sexual and asexual reproduction occur in two phylogenetically distant organisms and give rise to complex interactions between hosts, vectors, and parasites, while promoting constant coevolution between them^3,4^. In birds, *Plasmodium* infection is characterised by an acute phase, in which high parasite loads are reached soon after infection, followed by a chronic phase characterised by lower intensities that usually continue their course as lifelong infections^5^. Greater infection intensity has often been associated with a higher transmission rate^6^, but could reduce the transmission success of the parasite by killing either its vertebrate or invertebrate host^6^. Parasite virulence may thus be considered as a balance between increasing parasite transmission and reducing the costs imposed on their hosts^1^.

Different factors may influence the transmission success of vector-borne pathogens that cause major diseases, such as malaria, Lyme or bluetongue diseases^7–9^. Among them, the mosquito survival rate is particularly important^10^. Mosquitoes must be able to survive long enough for the *Plasmodium* sporozoites to develop (7–13 days^2,11^) to guarantee subsequent parasite transmission. Therefore, the lifespan of infected mosquitoes will have drastic consequences for the *Plasmodium* transmission success^12,13^. Avian malaria infections may impact the mosquito longevity^14^ (but see Pigeault and Villa^15^) and the development of parasites (i.e. mosquito latent period^16^). Although the impact of *Plasmodium* on the survival of birds has been experimentally demonstrated^17,18^, much less is known about the effects of infection on vector survival^16^. Mosquito survival may be reduced by *Plasmodium* due to tissue damage during the development and migration of parasites from the midgut to the salivary glands^19^ and the activation of a costly immune response against the infection^20^. However, positive^14^, negative^21^ and no effects^15^ of the infections by avian *Plasmodium* on mosquito survival have all been reported. The infection intensity in hosts may determine the successful development of the parasite in the mosquito^22,23^, although, in the case of *Plasmodium falciparum*, this relationship is not linear^24^. Likewise, a quadratic relation between parasitaemia in bird hosts and oocyst burden in mosquitoes has been found for the case of avian *Plasmodium*^25^.

Here, we used birds naturally infected by avian *Plasmodium* parasites to experimentally test the effect of the infection intensity of vertebrate hosts on mosquito survival, infection rate and *Plasmodium* transmission rate. *Culex pipiens*, the main vector of avian *Plasmodium*^2,26^, were allowed to bite *Plasmodium*-infected birds that had been either medicated with the antimalarial primaquine, which reduces infection intensity, or non-medicated control birds.

## Results

**Figure 1:**
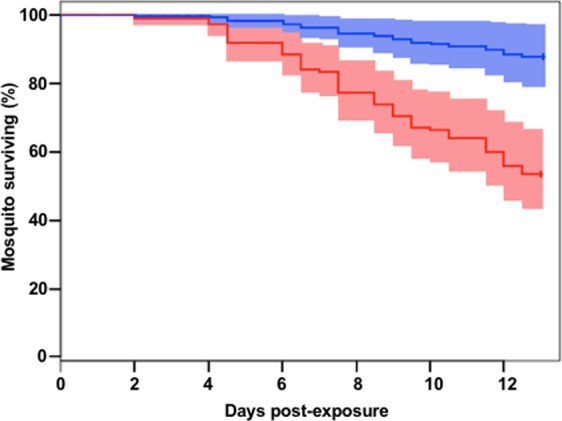
Percentage of mosquitoes’ survival until 13 days post-exposure to primaquine-medicated (blue) and control birds (red). The shaded areas comprise the standard errors.

The primaquine treatment significantly reduced the infection intensity in medicated birds compared to the controls (mean±SE control = 1.69 ± 0.26, medicated = 0.79 ± 0.23, F_1,35_ = 5.77, p = 0.02). Mosquitoes fed on birds infected by four different parasite lineages: *Plasmodium relictum* lineages SGS1 (18 birds) and GRW11 (3 birds), and the lineages PADOM02 (3 birds) and COLL1 (1 bird). The mosquitoes that fed on medicated birds (N = 102 mosquitoes) had a higher daily survival probability than those that fed on control birds (N = 95 mosquitoes) (daily survival probability = 0.99 and 0.96, respectively, Z = −3.17, p = 0.002; Table 1; Fig. 1). The presence of *Plasmodium* in the head-thoraxes of surviving mosquitoes was evaluated in 76 and 46 individuals that fed on medicated and control birds, respectively. *Plasmodium* was detected in 31 and 12 mosquitoes that had fed on medicated and control birds, respectively, and the presence of *Plasmodium* was screened in the saliva of these mosquitoes (Table 1). Overall, two out of 12 (16.67%) and 11 out of 31 (35.48%) head-thoraxes-positive mosquitoes were also positive in their saliva. The medication treatment did not affect the proportion of mosquitoes with *Plasmodium-*positive head-thorax (est = 0.89, Z = 0.92, p = 0.36, Table 1) or saliva (est = 1.31, Z = 1.66, p = 0.10, Table 1). *Plasmodium* lineages isolated from the head-thorax of mosquitoes and their saliva were identical. With the exception of the *Plasmodium* lineage COLL1, all *Plasmodium* lineages infecting house sparrows were isolated from mosquito saliva.

**Table 1:** Number of engorged, surviving and analysed *Culex pipiens* mosquitoes for the two experimental groups of birds (i.e. medicated and control). The number of Plasmodium positive/analysed head-thorax and mosquito saliva is given for each group. * Three mosquitoes fed on control birds and four mosquitoes fed on medicated birds escaped and were not included in survival analyses. ** Three mosquitoes fed on control birds and ten mosquitoes fed on medicated birds were not analysed due to logistical problems.

Treatment	Engorged mosquitoes*	Alive mosquitoes 13 days post exposure	Analysed mosquitoes**	Positive Head-thorax	Positive Saliva
Control	95	49	46	12	2
Medicated	102	86	76	31	11

## Discussion

The insect vector survival and infection rate may greatly affect the epidemiology of vector-borne parasites. Through an experimental manipulation of the infection intensity in wild birds, we assessed the impact of *Plasmodium* parasitaemia on mosquito survival, infection rate (i.e., presence of parasites in the head-thorax) and *Plasmodium* transmission rate (i.e., presence of parasites in the saliva). Medication reduced the infection intensity in birds, which in turn influenced mosquito survival, since higher *Plasmodium* intensities gave rise to greater mortality rates. Consequently, the mosquitoes that fed on medicated birds had a higher lifespan than those that fed on control birds. This result should be considered with caution as we estimated the survival of mosquitoes at only 13 days post-exposure and not along its complete lifetime. This period was selected based on the capacity of parasites to develop in mosquitoes between 7–13 days after infection^2^. The alternative interpretation that the greater survival rates in the mosquitoes that fed on medicated birds was in fact due to an effect of the drug itself on mosquito survival is poorly supported as the biological half-life of primaquine in plasma is 4–9 h^27^, and mosquitoes fed on birds 10 days after medication. Even if the drug had been active when ingested by the vectors, it could have not favoured mosquito survival^28^.

The costs of *Plasmodium* infection on mosquito survival are still a subject of intense debate^19,29^. Vézilier *et al*.^14^ reported increased longevity in *Cx*. *pipiens* infected by *P*. *relictum*; Pigeault and Villa^15^, by contrast, found that there were no effects on mosquito survival when using the same mosquito–parasite assemblage. However, these effects could be driven by the access to nutritional resources other than blood and the cost of *Plasmodium* infection on mosquito survival may only be detected in the event of nutritional stress^21^. The consumption of glucose has been found to be higher in infected mosquitoes than in uninfected ones^30^, which could be associated with the increased resources required by mosquitoes to fight off infections^31^. In addition, although Motta *et al*.^32^ did not find differences in the glucose concentration between *Plasmodium* infected and uninfected birds, avian malaria infection could affect the quality of the host blood through changes in the micronutrient composition and a decrease in red blood cell density. This potential decrease in host blood quality is expected to be a function of host parasitaemia^33,34^. Therefore, we cannot rule out the possibility that the observed lower survival rate of mosquitoes fed on control birds was due to the lower quality of these bloodmeals, and not exclusively to the costs of the parasite infections. However, since our mosquitoes were fed *ad libitum* with sugar solution after the bloodmeals, we suggest the lower survival of control mosquitoes was an effect of parasite infections and not due to the lower concentration of micronutrients in control birds’ blood. Parasites may impose additional costs on mosquitoes by producing tissue damage during their development, thereby increasing their susceptibility to bacterial infections and diseases^35^. An important factor likely affecting mosquito survival is the immune response of insects against parasite infection, which may also vary depending on the parasite species. For instance, Michel *et al*.^36^ found that the immune response of *Anopheles gambiae* performed differently against *Plasmodium falciparum* and *P*. *berghei*, which in turn affected the mosquito lifespan. The presence of four different *Plasmodium* lineages in our study could have potentially influenced our results on mosquito survival. This potential limitation is due to the fact that naturally infected birds were used in this experiment, thus providing an overview of the natural processes occurring in the wild. In fact, a diversity of parasite lineages circulate between birds and mosquitoes under natural conditions, with 12 different *Plasmodium* lineages infecting house sparrows in the study area^37^. Unfortunately, the sample size of individuals infected by each *Plasmodium* lineage was insufficient as to test for differences in the effect of the different parasite lineage on mosquito survival.

The infection intensity by *Plasmodium* might determine the success of parasite development in the insect vector and, consequently, its capacity for parasite transmission. In humans, the density of *Plasmodium* gametocytes has been found to be positively associated with the proportion of mosquitoes harbouring oocysts. However^23^, and in agreement with our findings, Pigeault *et al*.^25^ failed to find any association between avian *Plasmodium* infection intensity and the probability of mosquito infection. The absence of significant associations between the experimental reduction of *Plasmodium* infection intensity and the proportion of infected mosquitoes reported here could be due to the ability of *Plasmodium* to develop in mosquitoes that have fed on vertebrate hosts with infection intensities that are low or undetectable by microscopy^24,38^. This may also explain the absence of any significant effect of the reduction of infection intensity on the presence of *Plasmodium* in mosquito saliva 13 days after ingestion, a period which is enough for *Plasmodium* to reach the salivary glands^2,26,39^. However, the absence of significant differences could be due, at least in part, to the reduced sample size, as the prevalence was much higher in mosquitoes fed on control than on medicated birds (Table 1).

In wild bird populations, infections by avian *Plasmodium* usually pass through an acute phase of infection with high parasite loads followed by a chronic phase with low infection intensities^5^. Previous studies found that the prevalence of infection observed in mosquitoes fed on birds with acute infections was higher than in those mosquitoes fed on birds with chronic infections^25,40^. According to our results, and owing to the negative effects of high infection intensities on mosquito survival, *Plasmodium* transmission may be more effective during the chronic phase of infection than during the acute phase. Interestingly, Cornet *et al*.^39^ found that mosquitoes prefer biting avian hosts in a chronic phase of infection to biting uninfected birds or birds with infections in an acute phase, which is further support for how chronically infected birds affect the epidemiology of avian *Plasmodium*. Considering differences in host attraction by mosquitoes according to their infectious status and the impact on mosquito survival, as we did here, would provide a more realistic view of the epidemiology of avian malaria parasites in the wild.

## Material and Methods

### Mosquito collection and rearing

*Culex pipiens* larvae were collected in July 2014 in the natural reserve *La Cañada de los Pájaros* (Seville, Spain; 6°14′W, 36°57′N). Larvae were transferred to the laboratory and maintained in fresh water in plastic trays at uniform density, and fed *ad libitum* (Mikrozell 20 ml/22 g; Dohse Aquaristik GmbH & Co. KG, D-53501, Gelsdorf, Germany). Larvae and adult mosquitoes were maintained at 28 ± 1 °C, 65–70% relative humidity and 12:12 h light:dark cycle. Adult mosquitoes were anesthetised with ether, sexed and identified to species level under a stereo-microscope (Nikon SM7645) on chilled Petri dishes using morphological keys^41^. Female mosquitoes were placed in insect cages (BugDorm-43030F, 32.5 × 32.5 × 32.5 cm) and fed *ad libitum* with 1% sugar solution. Sugar solution was replaced with water 24 h prior to each experiment and the water was removed from the cages 12 hours before the experiment begins. The experiment was conducted using 7–15-days old female mosquitoes.

### Bird trapping and sampling

Yearling house sparrows (*Passer domesticus*) were captured using mist nets in July 2014 (6°50′W, 37°18′N). Birds were individually ringed and blood was sampled (0.2 ml) from the jugular vein using sterile syringes to assess their *Plasmodium* infection status using molecular methods (see details below). Birds were transported to the laboratory in the Doñana Biological Station (EBD-CSIC) and kept in birdcages (58.5 × 25 × 36 cm) in a vector-free room under controlled conditions (22 ± 1 °C, 40–50% RH and 17:7 h light:dark cycle). Birds were housed for 24 days before the exposing to mosquitoes and were fed *ad libitum* with a standard mixed diet for seed-eating and insectivorous birds (KIKI, GZM S.L., Alicante, Spain). It was not possible to discriminate the stage of *Plasmodium* infection (i.e. acute or chronic stage) of the birds. However, avian malaria infections reached its maximum after 10–13 days, while the chronic phase start after 20–25 days post infection^42^. Birds in our study were captured and maintained in a vector-free environment during 24 days before exposing them to mosquitoes, thus infections are expected to be in a chronic stage.

### Experimental procedure

The bird’ infection status was determined by the amplification and sequencing of a fragment of the parasite cytochrome b gene (see details below)^43^. Thirty-six house sparrows, naturally infected by *Plasmodium*, were randomly assigned to one of two experimental groups: medicated birds (the experimentally reduced infection intensity group, N = 17) or control birds (non-medicated group, N = 19). Medicated birds were injected subcutaneously with 0.1 mg of the antimalarial drug primaquine (Sigma, St. Louis, MO, USA) diluted in 0.1 ml saline solution while control birds were injected subcutaneously with the same volume of saline solution^44^. Primaquine was previously used to reduce the intensity of infection by avian malaria and malaria-like parasites in different bird species, including house sparrows^17,45,46^. In vertebrates, high doses of primaquine produces non-desirable side effects, such as gastrointestinal disturbances and the development of methaemoglobinaemia^47^. Thus, only a single and low–concentration dose of primaquine was administered to minimize these side effects. A single dose will clear most of the gametocytes within seven days after treatment, as reported in humans^48^. Ten days after the treatment, each bird was immobilized (using a cylinder of 1 × 1 cm mesh, allowing mosquitoes can bite through) and exposed individually to 80 unfed female *Cx*. *pipiens* in an insect cage (BugDorm-43030F 32.5 × 32.5 × 32.5 cm) for 30 minutes. Although previous studies allowed mosquitoes to fed on domestic birds during a longer period^40^, the duration of the exposure period was chosen to obtain a sufficient number of engorged mosquitoes while reducing stress levels experienced by wild birds, as those used in this study. Using this procedure, a mean of 8.5 mosquitoes fed on bird blood (range: 0 to 22 mosquitoes). This value is similar (mean = 14.2) to that obtained with *Cx*. *pipiens* allowed to feed on birds exposed overnight^44^.

Immediately after the trials, engorged mosquitoes fed on the same individual bird were captured and placed together in a single insect cage under the same standard conditions detailed above. Mosquitoes were fed *ad libitum* with 1% sugar solution. Mosquito survival was monitored every 12 h for 13 days post-exposure to allow for parasite development. At the end of this period, saliva from the surviving mosquitoes was obtained by introducing the mosquitoes’ proboscis into a 1 μl disposable capillary (Einmal-Kapillarpipetten, Hirschmann® Laborgeäte, Germany) with 1 μl of foetal bovine serum. One μl of 2% pilocarpine (Novartis 2012, Alcon Cusí S.A. Barcelona, Spain) was applied to the mosquito thorax to stimulate salivation. After 45 min, the medium containing the saliva was placed in 1.5 ml Eppendorf tubes with 10 μl of MQ water^26^. We chose the isolation of saliva over other conventional methods such as the analysis of mosquito salivary glands because it allows the use of molecular methods for parasite detection and it has been widely used in studies on the competence of mosquitoes to transmit pathogens, such as West Nile virus^49^, Dengue virus^50^, Zika virus^51^, human malaria parasites^52^ and avian malaria^26^. This method, however, required the mosquitoes to be alive, which implied stopping the monitoring of mosquito survival at 13 days post exposure. The alternative extraction of salivary glands may become difficult in dead mosquitoes because the tissues dry soon after death. Samples were kept at −80 °C until further molecular analyses.

One day after the trial, the birds’ blood was sampled again (0.2 ml) to confirm the blood parasite lineages infecting individuals at this stage. This procedure allowed us to identify any potential parasite lineage that was not detected during the first sampling. After sampling, a drop of blood was immediately smeared, air-dried, fixed in absolute methanol and stained with Giemsa for 45 min^53^. The intensity of infection by haemosporidian parasites was estimated as the percentage of infected red blood cells detected after scanning 10,000 erythrocytes from each blood smear at high magnification (x10,000). Birds were not blood-sampled immediately before or during the mosquito exposure period in order to reduce the stress caused by the blood extraction and mosquito bites. Birds were released after the completion of the experiments at the site of capture.

### Molecular detection and identification of blood parasites

DNA was isolated from blood samples and the head-thorax of each mosquito using a semiautomatic procedure (MAXWELL® 16 LEV Blood DNA Kit)^54^. The Qiagen DNeasy® Kit Tissue and Blood (Qiagen, Hilden, Germany) was used to isolate DNA from mosquito saliva. Based on a previous study showing that saliva from uninfected mosquitoes tested negative^26^, we considered absence of *Plasmodium* DNA in the saliva of those mosquitoes with uninfected head-thoraxes. Therefore, we only analysed those saliva samples from mosquitoes with *Plasmodium* positive head-thoraxes. *Plasmodium* infections were recorded following Hellgren *et al*.^43^. The presence of amplicons was verified in 1.8% agarose gels and positive samples were sequenced using the Macrogen sequencing service (Macrogen Inc., Amsterdam, The Netherlands). Sequences were edited using the software *Sequencher*™ v 4.9 (Gene Codes Corp., © 1991–2009, Ann Arbor, MI 48108) and assigned to parasite lineages through blast comparison with those deposited in the GenBank (National Center for Biotechnology Information) and Malavi databases^55^.

### Statistical analyses

An ANOVA test was used to assess differences in the *Plasmodium* infection intensity (log-transformed) between medicated and control birds. We fitted a Cox mixed-effect model by maximum likelihood to mosquito survival data (number of surviving mosquitoes /12-hours-period) to test the effect of counted the medication treatment on mosquito survival. The medication treatment was considered as a fixed factor, using censored survival data and bird identity as a random or ‘frailty’ effect. Two similar Generalized Mixed Linear Models (GLMMs) with binomial error and logit link function were performed in which the infection status by *Plasmodium* of the head-thorax or the saliva samples were included as the dependent variable, respectively. The medication treatment was included as a fixed factor and bird identity as a random term. Initially, 36 birds were included in the study comprising 17 medicated birds and 19 control birds. However, six of these birds, including two medicated birds and four controls, showed evidence of co-infections. To avoid any potential effect of parasite coinfection on mosquito survival^56^, those insects fed on co-infected birds (n = 51) were excluded from the analyses. Thus, the final sample size included 30 birds comprising 15 medicated birds and 15 controls. Mosquitoes fed on blood from all birds but two medicated and three control birds. Statistical analyses were performed in R software 3.2.5 (R Core Development Team, 2016) with the packages *survival*^57^ and *lme4*^58^.

### Ethics statements

All experiments involving animals adhered to the guidelines included in the Spanish Legislative Decree “Real Decreto 53/2013 de 1 de Febrero” on protection of animals used for experimentation and other scientific purposes, with the guidelines established by the European Community Council Directive n° 2010/63/UE on Laboratory Animal Protection. The project was approved by the Regional Authorities and the CSIC Ethics Committee (project code assigned by the CSIC Ethical Committee CEBA-EBD-12-40).

## Supplementary information

Dataset1

Dataset2

Dataset3

## Data Availability

The data generated and/or analysed during the current study are available from the corresponding author on reasonable request. Supplementary material are included in the manuscript.
